# Puumala Virus Variants Circulating in Forests of Ardennes, France: Ten Years of Genetic Evolution

**DOI:** 10.3390/pathogens10091164

**Published:** 2021-09-09

**Authors:** Guillaume Castel, Elodie Monchatre-Leroy, Marc López-Roig, Séverine Murri, Mathilde Couteaudier, Franck Boué, Denis Augot, Frank Sauvage, Dominique Pontier, Viviane Hénaux, Philippe Marianneau, Jordi Serra-Cobo, Noël Tordo

**Affiliations:** 1 CBGP, INRAE, CIRAD, IRD, Montpellier SupAgro, Université Montpellier, 34000 Montpellier, France; 2 Nancy Laboratory for Rabies and Wildlife, ANSES, 54220 Malzeville, France; denis.augot@anses.fr; 3 Departament de Biologia Evolutiva, Ecologia i Ciències Ambientals, Facultat de Biologia, Universitat de Barcelona, 08028 Barcelona, Spain; mlroig@gmail.com (M.L.-R.); serracobo@areambiental.com (J.S.-C.); 4 Institut de Recerca de la Biodiversitat (IRBio), Faculty of Biology, University of Barcelona, 08028 Barcelona, Spain; 5 Lyon Laboratory, ANSES, Virology Unit, University of Lyon, 69007 Lyon, France; severine.murri@anses.fr (S.M.); philippe.marianneau@inrae.fr (P.M.); 6 INSERM U1259 MAVIVH, Université de Tours and CHRU de Tours, 37032 Tours, France; mathilde.couteaudier@univ-tours.fr; 7 Nancy Laboratory for Rabies and Wildlife, ANSES, SEEpiAS Unit, 54220 Malzéville, France; franck.boue@anses.fr; 8 USC Vecpar, ANSES-LSA, EA 7510, Université de Reims Champagne-Ardenne, SFR Cap Santé, Faculté de Pharmacie, 51096 Reims, France; 9 SEENOVATE, 69002 Lyon, France; sauvage_frank@yahoo.fr; 10 UMR–CNRS 5558 Biométrie et Biologie Evolutive, Université C. Bernard Lyon-1, 69622 Villeurbanne, France; dominique.pontier@univ-lyon1.fr; 11 LabEx Ecofect, Eco-Evolutionary Dynamics of Infectious Diseases, University of Lyon, 69622 Lyon, France; 12 Lyon Laboratory, ANSES, Epidemiology and support to Surveillance Unit, University of Lyon, 69007 Lyon, France; viviane.henaux@anses.fr; 13 Institut Pasteur, Antiviral Strategies Unit, Department of Virology, 75015 Paris, France; ntordo@pasteur.fr; 14 Institut Pasteur de Guinée, Conakry BP 4416, Guinea

**Keywords:** *Orthohantavirus*, *Myodes glareolus*, seroprevalence, microdiversity, evolution, population dynamics

## Abstract

In Europe, Puumala virus (PUUV) transmitted by the bank vole (*Myodes glareolus*) is the causative agent of nephropathia epidemica (NE), a mild form of haemorrhagic fever with renal syndrome. In France, very little is known about the spatial and temporal variability of the virus circulating within bank vole populations. The present study involved monitoring of bank vole population dynamics and PUUV microdiversity over a ten-year period (2000–2009) in two forests of the Ardennes region: Elan and Croix-Scaille. Ardennes region is characterised by different environmental conditions associated with different NE epidemiology. Bank vole density and population parameters were estimated using the capture/marking/recapture method, and blood samples were collected to monitor the overall seroprevalence of PUUV in rodent populations. Phylogenetic analyses of fifty-five sequences were performed to illustrate the genetic diversity of PUUV variants between forests. The pattern of the two forests differed clearly. In the Elan forest, the rodent survival was higher, and this limited turn-over resulted in a lower seroprevalence and diversity of PUUV sequences than in the Croix-Scaille forest. Uncovering the links between host dynamics and virus microevolution is improving our understanding of PUUV distribution in rodents and the NE risk.

## 1. Introduction

Within the family *Hantaviridae* (Order *Bunyavirales*), the genus *Orthohantavirus* consists of viruses transmitted to humans via contaminated aerosolised excreta of small rodents. Hantaviruses are distributed worldwide, except in Antarctica, and are closely associated with their mammal vector, in which they do not show any obvious pathogenicity [1]. When transmitted to humans, they cause haemorrhagic fever with renal syndrome (HFRS) or Hantavirus cardio-pulmonary syndrome (HCPS), mainly depending on whether their rodent vector is located in the Old World (Europe, Asia) or in the New World (Americas) [2]. The pathogenicity of the infection, from asymptomatic to serious disease, is modulated by host immune defences [3,4] and depends on the potential of each viral variant to counteract these defences [5]. In the last few decades, hantaviruses have become emerging zoonotic pathogens, and the associated diseases have generated growing public health concern because of their increasing frequency, amplitude, and geographic expansion [2].

In Europe, *Puumala virus* (PUUV) is the agent of nephropathia epidemica (NE), a mild form of HFRS [6]. It is transmitted by the bank vole (*Myodes glareolus*), which is distributed throughout a large part of Europe. However, the spatial distribution of NE incidence is substantially smaller, with high variation at the geographical scale [7], and does not comply with the spatial distribution of the bank vole population or rodent infection density. In France, infected rodents are not only found inside the human endemic region [8] but are also found outside, without associated human cases [9]. A lack of awareness and misdiagnosis of some human cases are not sufficient to explain this discrepancy between the distribution of the infected reservoir and associated human disease, and the reasons have not been completely elucidated [7]. 

PUUV phylogeny presents strong geographical clustering at a large [10] and small scale [11], and the geographic clusters remain stable over time [11]. The main mechanisms driving PUUV genetic evolution are genetic drift, i.e., accumulation of nucleotide substitutions and small insertions/deletions [12,13,14,15], or genetic shift, i.e., reassortments between genome segments [15,16]. The microevolution of closely related viruses of the same viral species circulating in different geographic areas has already been observed in other viral models, such as rabies, and this phenomenon is still poorly understood [17]. Five-year monitoring of PUUV microevolution in bank vole populations in central Finland showed a quasi-neutral mode of evolution, with preservation of a few dominant genetic variants over several seasons and years. However, several nucleotide substitutions also indicated rapid adaptation of transient variants to environmental changes and new stressors [18]. These stressors are of multiple origins and include the immune system of individual bank voles, modulated by their age and reproductive and health status [19,20]. Moreover, the dynamics of the rodent population, including age structure and number of reproductive adults, fluctuates seasonally and annually [21] depending on climate and environmental conditions, including landscape and habitat frequently shaped by anthropic intervention. This can directly influence orthohantavirus circulation within their reservoir host and subsequent transmission to humans [22,23,24]. Harsh winters can drastically decrease the rodent population, resulting in a genetic bottleneck, with the extinction of some genetic variants of PUUV and the selection of others capable of infecting rodent survivors [25]. In this sense, population dynamics of rodents highly affect the genetic diversity of PUUV, and it is therefore of tremendous importance to follow orthohantavirus dynamics and evolution in changing environments.

In France, the NE endemic area is located in the northeast—in particular, in the Ardennes region. This region accounted for 30% to 40% of the reported annual cases over the 1996–2005 period [26,27,28] and for 7% to 26% over the 2012–2016 period [29]. However, the spatial distribution of human cases is heterogeneous. For example, the Croix-Scaille forest is a large spruce forest where many human NE cases have been reported, whereas Elan forest is a small hedge, broadleaf forest not linked to known human NE cases [8]. In both forests, rodents are infected [8]. In the present study, we combined capture/marking/recapture monitoring of the rodent population in these two forests from 2000 to 2009 and phylogenetic analyses to evaluate infected rodent population dynamics and viral microevolution of PUUV in bank vole populations.

## 2. Results

### 2.1. PUUV Microevolution 

#### 2.1.1. Phylogenetic Analysis

**Figure 1:**
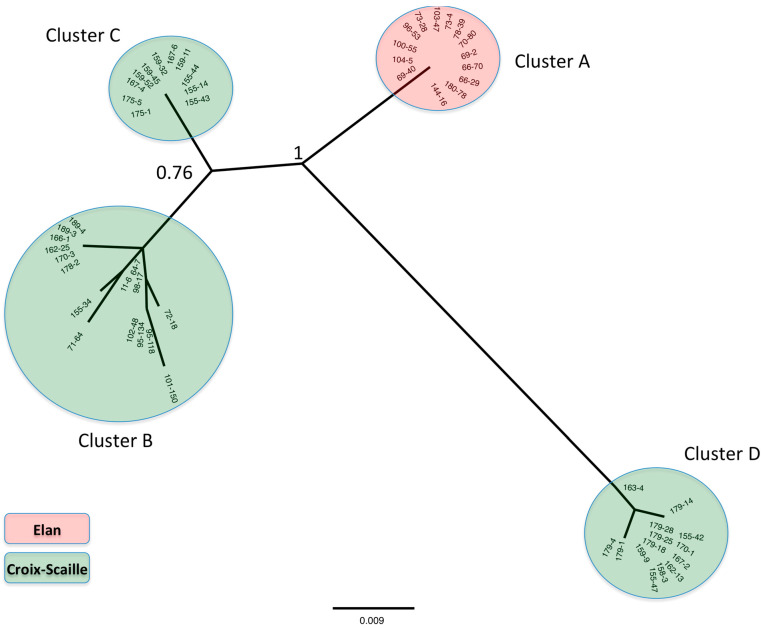
Unrooted phylogenetic tree based on the S segment (354–654 nt) of 55 PUUV isolates constructed using the ML method and HKY85 + I substitution model. Bootstrap percentages from 1000 resamplings are indicated at the two main nodes. Clusters of sequences from Elan (A) and Croix-Scaille (B, C, D) forests are in red and green, respectively. The scale bar indicates nucleotide substitution per site.

The final dataset consisted of 55 sequences that were deposited in GenBank. Accession numbers, sampling year, and station are indicated in Table S1. At the small geographical scale of these two forests, after phylogenetic analysis using the maximum likelihood (ML) method, we observed strong geographical clustering of the phylogenetic tree. Three clusters of genetic variants (B, C, and D) were shown to circulate in Croix-Scaille forest, and only one (A) in Elan forest (Figure 1). 

#### 2.1.2. Genetic Diversity of PUUV Isolates

Analyses of the overall mean distance of the 55 sequences showed that the average evolutionary divergence between all PUUV viruses circulating at the four stations was 0.037 base substitutions per site. All the mutations were silent, reflecting a strong purifying selection in this fragment of the coding region of the nucleoprotein gene. 

Combined analysis of the phylogenetic tree (Figure 1) and the average evolutionary divergence (Table 1) showed that isolates from Elan forest stations (3 and 4) had no genetic diversity (0% divergence/0 base difference) at least for the considered fragment, which is statistically different from those of Croix-Scaille forest stations, which presented far higher genetic diversity (19.3 base differences per sequence, corresponding to 3.2% divergence; χ2 = 19.6, *p* < 0.001). There was no statistically significant difference (χ2 = 0.27, *p* > 0.05) between genetic diversities at station 2 and station 5, which showed 3.5% and 2.8% base difference, respectively (Table 1).

#### 2.1.3. Evolution of Genetic Diversity over Time

**Figure 2:**
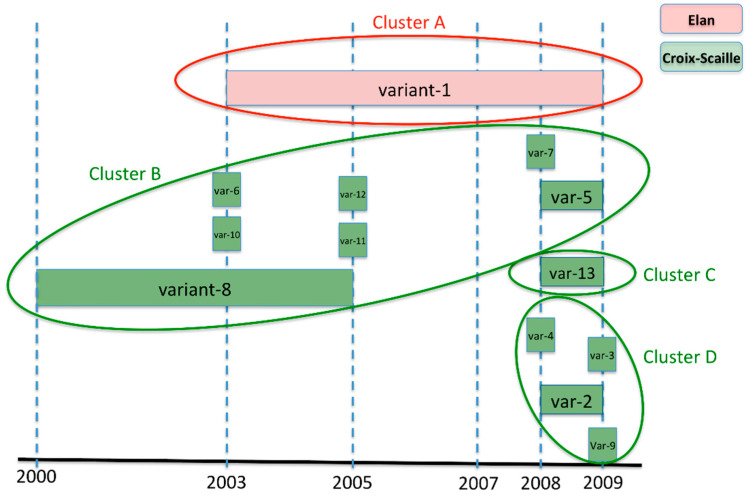
Local dynamics of PUUV evolution characterised by the emergence of different genetic variants over time at the different stations. Clusters A to D are the same as shown in Figure 1. Corresponding variant numbers for each sequence are indicated in Figure S1. All PUUV recovered from Elan forest from 2003 to 2009 (in red) correspond to the same genetic variant 1 (Cluster A) regardless of the trapping station (station 3 and station 4). Genetic variants from the Croix-Scaille forest (in green) were more numerous and more transient from 2000 to 2009. Cluster B: variants 7, 10, 11, and 12 were isolated at station 2 only, variants 5 and 6 at station 5 only, and variant 8 at both stations 2 and 5. Cluster C: variant 13 was isolated at station 2. Cluster D: variants 9, 3, and 4 were isolated at station 2 and variant 2 at both stations 2 and 5.

Figure 2 shows diversification over time of PUUV variants at the stations that are summarised in Table 2. While stations 3 and 4 in Elan forest kept exactly the same variant for 7 years (2003–2009), isolates from clusters B and D in Croix-Scaille forest were spread between stations 2 and 5 and showed more diversification over time: seven genetic variants were detected over 10 years within cluster B, and four genetic variants in only 2 years within cluster D. Isolates of cluster C were specific to station 2 (Table S1); interestingly they showed no diversification over their 2-year detection period (Figure 2).

### 2.2. Population Dynamics of Rodents

During the 10-year study period (2000–2009), we captured and identified a total of 2005 individual bank voles: 691 in Croix-Scaille forest (524 at station 2; 167 at station 5) and 1314 in Elan forest (771 at station 3; 543 at station 4).

**Figure 3:**
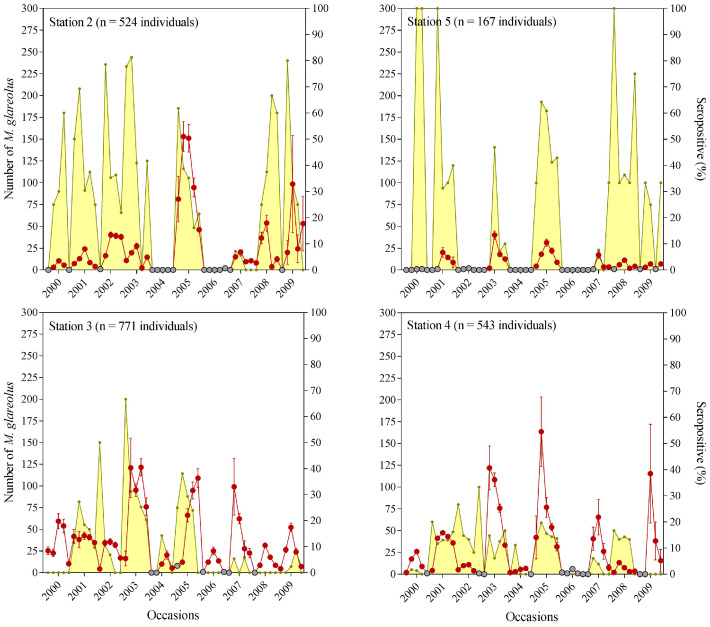
Estimated abundance of bank vole populations and observed PUUV seroprevalence at stations 2–5 (Croix-Scaille) and 3–4 (Elan): Left *y* axis: abundance of bank voles estimated by capture-recapture (red dots) and abundance not estimated (grey dots) when too few bank voles were trapped. Right *y* axis: seroprevalence (yellow) in percentage. n indicates the number of bank vole individuals captured at each station.

Closed-model estimates of bank vole population size showed high fluctuations throughout the study period (Figure 3). In Elan, the presence of bank voles was observed in every study year (except at station 4 in 2006 due to clear-cutting of trees at the trapping site). A similar pattern was observed at both stations, with remarkable peaks of abundance in 2003, 2005, and 2007 for both stations, and 2009 for station 4 (Figure 3). In contrast, the two stations in Croix-Scaille showed years with no (or too few) captures to estimate abundance of bank voles: 2004 and 2006 at the two stations and an additional year for station 5 (2002). Apart from these years, station 5 maintained constant low abundance, while station 2 showed peaks of abundance in 2005 and 2009 (Figure 3).

The most parsimonious models for apparent survival and seniority probabilities of bank voles were well supported with ΔAIC > 2 and a better Akaike weight (>0.70) compared with other models (Table S2). The variance inflation factor obtained from the bootstrap procedure indicated a reasonable fit to a general model. However, we used QAICc, adjusted by ĉ = 1.83, to account for overdispersion in our results for model selection, reported adjusted standard errors, and seniority probabilities. 

The Cormack-Jolly-Seber model indicated that annual survival probabilities (i.e., the probability that an individual alive in year *t* survived to the next year and remained in the study area) were constant in time for each station but differed between stations, ranging from 0.09 at station 5 to 0.31 at station 4 (Table 3). The model with different survival probabilities between stations was statistically different from the model with constant survival (χ2 = 8.18, *df* = 3, *p* = 0.042, Table S2). Despite the differences in survival probabilities between stations, we did not find significant differences when compared with pairs (only marginal significance between S4 and S5: χ2 = 3.78, *df* = 1, *p* = 0.052). The Pradel model selection also indicated significant differences between sites but no time variation in the seniority probabilities (i.e., the probability for an individual present at a given occasion to already be present at the previous occasion), which ranged from 0.07 (station 5) to 0.27 (station 4) (Table 3). Although all stations showed an increase in net population growth each year, the recruitment parameters (f, percentage of new migrant individuals at i + 1 per individual present at i) varied, indicating lower recruitment rates in Elan (especially at station 4) than in Croix-Scaille (Table 3). 

## 3. Discussion

This study presents ten-year monitoring data for host population dynamics, PUUV seroprevalence, and PUUV genetic diversity in two bank vole populations living in two forests of the Ardennes region with different environmental conditions. We show that PUUV microevolution, population dynamics, and seroprevalence of bank voles display different patterns between forests (Table 4). In Elan forest, the study highlighted lesser diversity of PUUV sequences, a higher rodent survival, a more continuous rodent presence, and a lower seroprevalence than in Croix-Scaille forest. It is obvious that the limited genomic region (a 303 nucleotides fragment of the S segment) used for genetic analysis is not the most suitable for solid phylogenetic data. This limitation is explained by the original design of this study aiming to compare rodent seroprevalence between areas with different NE epidemiology [8]. The genomic analysis later emerged in complement because of the interest in the serological results. In addition, the sampling by capture/release/recapture allowed only a limited volume of blood to be taken (100 µL of blood per rodent), and most of it was consumed by the serological studies. Nevertheless the genetic data are associated with demographic data collected during ten years using capture/marking/recapture method and analysed with a robust design, known to be more biologically realistic [30]. Altogether, this provides research hypotheses based on a multifactorial approach to the virus–host ecosystem. Further studies based on complete PUUV genome sequences will offer an opportunity to challenge them in the future.

Although all genetic variants belong to the Central European lineage [9], we clearly showed PUUV genetic diversity, which may even be underestimated because the targeted sequence is in the conserved central domain of the N protein [31,32]. This domain is under strong genetic constraints [31], explaining that no amino acid variation was observed. However, there was still enough evolution at the nucleotide level to provide reliable differences between stations. Importantly, stations 2 and 5 in Croix-Scaille forest showed stronger PUUV diversification than stations 3 and 4 in Elan forest which maintained the same variant across the study. Previous local-scale studies have shown that maintenance of preferred genotypes or lineage turnover can occur depending on the land cover type [18,33], and several hypotheses described below linked to the environment impacting rodent population dynamics may explain the differences in PUUV diversity between stations. 

Elan forest (stations 3 and 4) showed persistence of a single genetic variant from 2003 to 2009. With its small size and being surrounded by fields and roads, Elan forest probably favoured the isolation of bank vole populations and limited the number of circulating PUUV genotypes, although such physical obstacles are not absolute barriers in the Ardennes [33]. The sustainable settlement of bank voles allowed by good environmental conditions prevented the collapse of the rodent populations and of their associated PUUV variant. 

In contrast to Elan forest, our results show that PUUV genetic diversity in Croix-Scaille forest (stations 2 and 5) was high over time. Environmental conditions at station 5, with harsher weather conditions due to the higher altitude of 503 m above sea level (a.s.l) and conifers, were the least favourable for rodents, as shown by the lowest survival predicted by our model, numerous extinctions of population, and low abundance. Given that station 5 is in a large forest, population persistence likely resulted from the recruitment of bank voles emigrating from surrounding sites. This kind an asymmetrical bank vole migration from a large forest to hedge wood was also observed in another study [34]. Therefore, the high PUUV diversity at station 5 was associated with emigrating and surviving bank voles, potentially infected with different viral variants. Another hypothesis can be given for station 2, at which PUUV diversity was the highest. Despite similar weather conditions to station 5, plots with oaks were more favourable, as shown by higher survival and abundance of bank voles. In Western Europe, the preferred habitat for voles is deciduous forest [35]. The diversity of PUUV at station 2 could thus be due to easier accessibility to migration, with better conditions for rodent survival. This hypothesis is supported by the relationship in station 2 over time between strain diversity and rodent abundance. Our results show more rodents and more viral diversity at station 2 in 2005 and 2009 and to a lesser extent in 2003.

The evolution of seroprevalence over time also differed between sites. In Elan forest, seroprevalence was found to be low to moderate (<30%), with annual fluctuations at station 4, while it reached 40% to 60% in 2002, 2003, and 2005 at station 3. In contrast, the level of seroprevalence in captured animals in Croix-Scaille forest was clearly higher (>60%) than in Elan forest, almost over the full duration of the study. These differences in seroprevalence between sites may be associated with different PUUV microevolution patterns under different environmental conditions. Weather conditions with lower temperatures and higher moisture levels, such as in Croix-Scaille forest, enhance PUUV survival in the environment and potentially increase rodent contamination [36,37]. Moreover, limited food resources and difficult weather conditions can decrease bank vole immune response, as it can in other rodents [38,39], which may also be potentially impacted by new viral variants with different phenotypical properties. Several studies have explored the effect of microevolution on virus phenotype. For example, different replicative properties were observed between wild-type and Vero E6 cell-cultured variants of PUUV, with only one nucleotide mutation in the non-coding region [40]. Another study highlighted the fixation of one silent mutation during in vivo transmission of PUUV, suggesting an advantage for viral transmission [41]. Higher genetic PUUV diversity and harsher environmental conditions, as observed in Croix-Scaille forest, can result in the host immune system having lower control over viral replication and greater viral excretion dynamics [25,33], as well as potential higher seroprevalence. 

Finally, Elan forest and Croix-Scaille forest were originally selected [8] because they were associated with few to numerous human cases in the early 2000s, respectively. Our results and the associated implications on rodent contamination levels may explain the differences in human contamination rates between these two forests. The suspected higher viral excretion by bank voles in Croix-Scaille forest may have resulted in sufficient environmental contamination to enhance human infections. Our field results are consistent with this hypothesis and underline the potential role of PUUV variants. It is interesting to note that during 2005 and 2007, the proportion of infected bank voles was higher in Croix-Scaille than in Elan forest. These years corresponded to records for human infections in the Ardennes [42]. Therefore, regular monitoring of rodent abundance, of virus prevalence, and of PUUV microevolution at sites associated with numerous human cases (like Croix-Scaille) would allow for better prediction and ideally better prevention of human infections [11].

The discovery of NE cases outside the classical area of NE distribution in France in more western and southern areas [43] emphasizes the need for a better understanding of the mechanisms leading to human infections. This study showed several differences between sites with numerous or few human cases, suggesting that PUUV microevolution is associated with rodent population dynamics and the environment. Up to now, the influence of PUUV diversity in such systems has been studied less closely than the other components. Ecological knowledge on PUUV and on the impact of viral diversity on rodent infection should be taken into account to improve prediction of human risk [37]. Assessment of PUUV distribution and diversity in France and in Europe should also be considered since the strong geographical clustering of PUUV isolates at small and large geographical scales allows us to identify the most likely places for PUUV-infected patients [11,44]. 

## 4. Materials and Methods

### 4.1. Rodent Trapping and Collection Data

**Figure 4:**
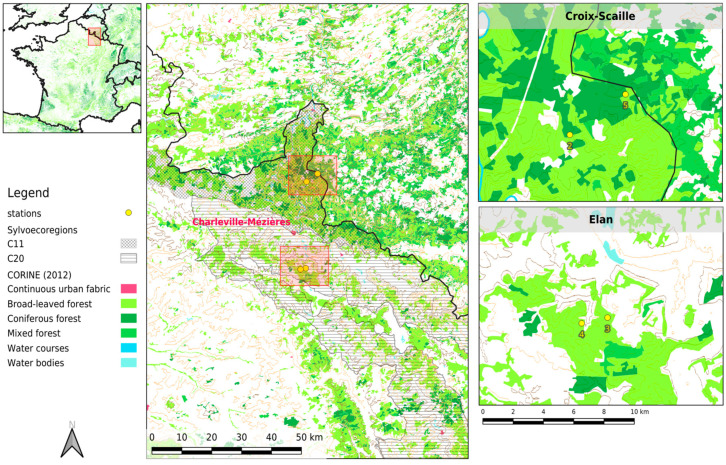
Localisation of the sampling stations. This map was produced with QGis 3.16 using Corine Land Cover (CLC) 2012 data, (Version 2020_20u1, available from https://land.copernicus.eu/pan-european/corine-land-cover/clc-2012?tab=metadata, accessed on 27 August 2021) and data from the Inventaire Forestier National, (available from https://inventaire-forestier.ign.fr/spip.php?article532, accessed on 27 August 2021). Ardennes sylvoecoregions (areas with specific original forest habitat) C11 (Primary Ardennes) and C20 (Northeast limestone plateaux) are shown. Geographic coordinates of the four stations are: Station 2 (49°55′18..66′′ N; 4°47′15.05′′ E), Station 3 (49°39′44.11′′ N; 4°46′27.46′′ E), Station 4 (49°39′33.24′′ N; 4°45′1.93′′ E), and Station 5 (49°56′42.32′′ N; 4°50′22.22′′ E).

This study was the continuation of the Augot et al. study [8]. The four sampling sites were the same, with two stations in Elan forest (stations A and B, renamed 3 and 4 here, respectively) and two other stations in Croix-Scaille forest (stations C and D, renamed 2 and 5 here, respectively). They are separated by the city of Charleville-Mézières (Figure 4). Although only 30 km apart, these two forests belong to two different “sylvoecoregions” with different weather and environmental conditions. Stations 3 and 4, on the Northeast limestone plateau, are 2 km apart and are located in a limited forest of broadleaves (mainly beeches, accompanied by oaks and charms) surrounded by fields and roads. Stations 2 and 5, in the primary Ardennes, are 5 km apart and are located in a large forest massif, mainly consisting of conifers at station 5 and oaks with conifers at station 2, with a few small clearings where villages are located. The altitude in Croix-Scaille is higher (503 m a.s.l) than in Elan (186 m a.s.l). Weather conditions are also harsher in Croix-Scaille, with lower average temperatures (2.2 °C, comparison of means by Student’s *t* test: *p* < 0.05) and amounts of monthly precipitation (24.3 mm, comparison of means by Student’s *t* test: *p* < 0.001). Late frosts in May are common, and temperatures may drop below zero in June or September at this station. It rains or snows on an average of 180 to 190 days a year, with annual precipitation from 1190 to 1300 mm. 

**Figure 5:**
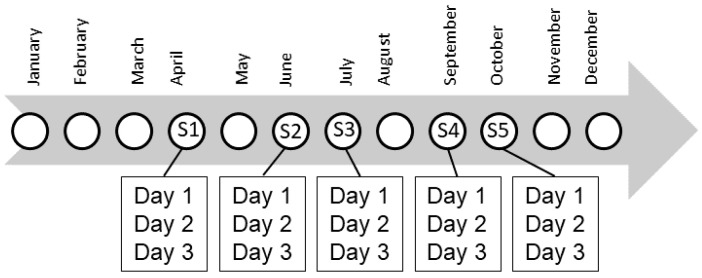
Annual sampling design with five primary trapping sessions in April, June, July, September, and October: S1 to S5. For each session, three successive trapping days were performed. Depending on the years, the five trapping sessions could also concern August instead of July.

The trapping protocol for each station consisted of one plot with a trap grid of 49 (7 × 7) Ugglan live traps separated from each other by a distance of 14 m. Traps were deployed for three successive nights. This methodology was used in five trapping sessions during the most active season for *Myodes glareolus* (April, June, and July or August, September, and October) for each year from 2000 to 2009 (10 years). For each trapping session, there were three consecutive trapping days (Figure 5).

Captured rodents were identified with the taxonomic key of Quéré and Le Louarn [35], weighing, sexed, and marked by toe-clipping before being released at their original site of capture. Blood was taken from the retro orbital sinus without anaesthesia. All procedures complied with EC Directive 86/609/EEC and its French transposition (Decree 2001-486, June 2001), which were in force during the study.

The collected blood samples were stored on field in a cooler at +4 °C. They were centrifugated (10,000 RPM for 5 min) on return to the laboratory (maximum storage time in the cooler of 3 days), and the resulting sera were stored at −20 °C.

### 4.2. Serological and Molecular Analysis

Bank vole serum samples were screened for previous PUUV exposure by an IgG ELISA assay on plates coated with N recombinant protein of PUUV or controls, as described in Castel et al. (2015) [9].

Seroprevalence was calculated as the proportion of PUUV-seropositive rodents among all bank voles trapped for each monthly session. All individuals weighing less than 14 g, considered young individuals still protected by maternal antibodies, were excluded. 

RNA was extracted from 55 serum samples of seropositive bank voles for which the quantity of RNA was sufficient, using a QIAamp viral RNA extraction kit (Qiagen), following the manufacturer’s recommendations. Reverse transcription-PCR (RT-PCR) was performed essentially as described earlier in Plyusnina [45]. Sequences of primers are available upon request. PCR-amplicons were purified from agarose gel and sequenced by the Sanger method (nucleotides 352–654 of the coding part of the PUUV S segment; 101 aa).

### 4.3. PUUV Microevolution 

Multiple sequence alignments were prepared with the Clustal Omega alignment programme, implemented in SEAVIEW v4.6.1 [46]. Phylogenetic reconstructions were conducted with the maximum likelihood (ML) approach using PhyML v3.1, implemented in SEAVIEW v4.6.1. The optimal substitution model was identified as the HKY85 + I (0.79) model using the SMS v1.8.1 programme [47], available online at http://www.atgc-montpellier.fr/sms/ (accessed on 1 January 2020) on the ATGC bioinformatics platform. The transition/transversion ratio was fixed to 4, and nucleotide frequencies were optimised from the data set. Support for individual nodes was assessed using an approximate likelihood ratio test (aLRT) implemented in PhyML v3.1. Phylogenetic trees were visualised using FigTree v1.4.3. The estimate of genetic divergence at the nucleotide scale within and between stations was calculated using a function implemented in the Mega v7.0 programme. Analyses were carried out using maximum composite likelihood. The rate variation among sites was modelled with a gamma distribution (shape parameter = 1). All the other parameters were set to their default value. All ambiguous positions were removed for each sequence pair.

### 4.4. Bank Vole Population Dynamics

To estimate population size for each period, we used the robust-design model within the MARK programme [48], which combines the Cormack–Jolly–Seber model [49,50,51] and closed-capture models [52,53]. The robust design consists of the five primary trapping periods (April, June, and July or August, September, and October), over which populations are open. For each primary trapping session, three consecutive trapping days were performed, over which populations are assumed to be closed (Figure 5). This design allowed us to use historical encounter input data for the robust-design models, to estimate capture and recapture probabilities, and subsequently to improve the precision of population size estimates [53]. No goodness-of-fit tests are available for robust-design models [54]. Bank vole population size was not estimated when no (or too few) captures were obtained in trapping days or months. In these cases, the number of captures obtained on each occasion was considered.

We also used the Cormack–Jolly–Seber and Pradel models within the MARK programme to estimate annual apparent survival (i.e., the probability that an individual alive in year *t* survived to the next year and remained in the study area) and seniority probabilities (i.e., the probability for an individual present at a given occasion to already be present at the previous occasion) of bank voles, respectively. Time (in years within the 2000–2009 period), site (four sites: 2–5), and interaction between time and site were included to generate 16 different candidate models including all possible combinations. We used the bootstrap procedure (Mark programme) to test the goodness-of-fit, and we used the variance inflation factor ĉ (deviance / mean / Dev), estimated to correct for the overdispersion in the data, before model selection if necessary (White and Burnham 1999). We used the Akaike’s information criterion (as the likelihood of the model given the model set) corrected for small sample size: AICc or QAICc (when the overdispersion is corrected for with the factor ĉ) to select the most parsimonious model. The model with the smallest AICc value was selected as the best supported model when its AICc difference with other models (ΔAICc) exceeded two [55]. Differences in survival probabilities among the three stations were tested with a generalised chi-square statistic [56] available in CONTRAST [57]. 

Finally, we estimated the population rate of change (λ) and per capita recruitment rate (*f*) from the Pradel formulation [58]. The net population rate of change (*λ*i) between survey *i* and *i* + 1 is given by:

*λi* = *ϕi*/*Υi* + 1 = *ϕi* + *fi*,

where *ϕi* and *Υi* + 1 are the apparent survival rate and the seniority probability, respectively, and *fi* is the per capita recruitment rate (percentage of new migrant individuals at *i* + 1 per individuals present at *i*).

## Figures and Tables

**Table 1:** Estimates of average evolutionary divergence over sequence pairs: within-station in grey boxes and between-stations in white boxes. Stations 2 and 5 are in Croix-Scaille forest and stations 3 and 4 in Elan forest. The average number of base differences (and the corresponding percentages) are shown. The analysis involved 55 nucleotide sequences of 303 nucleotides long each, after removal of all ambiguous positions. Codon positions included were 1st + 2nd + 3rd... Evolutionary analyses were conducted in MEGA7. ND: not done.

	Station 2	Station 3	Station 4	Station 5	Elan Forest	Croix-Scaille Forest
**Station 2**	10.75 (3.5)	12.67 (4.2)	12.67 (4.2)	10.62 (3.5)	ND	ND
**Station 3**		0 (0)	0 (0)	12.64 (4.2)	ND	ND
**Station 4**			0 (0)	12.64 (4.2)	ND	ND
**Station 5**				8.51 (2.8)	ND	ND
**Elan forest**					0 (0)	ND
**Croix-Scaille forest**						19.3 (3.2)

**Table 2:** Presence and diversification of the four main sub-lineages isolated in Elan and Croix-Scaille forests over time. NO: not observed.

Station (Forest)	Detection Period	Diversification (Number of Different Variants for Each Cluster)				Total Number of Different Variants for Each Station
		Cluster A	Cluster B	Cluster C	Cluster D	
3 (Elan)	2003–2009	1	NO	NO	NO	1
4 (Elan)	2003–2005	1	NO	NO	NO	1
2 (Croix-Scaille)	2000–2009	NO	5	1	4	10
5 (Croix-Scaille)	2003–2009	NO	3	0	1	4

**Table 3:** Demographic parameters per station.

Station	Survival (*ϕ*) ^a^	Seniority (*Υ*) ^a^	Recruitment (*f*) ^b^	Population Growth (*λ*) ^b^
**Croix-Scaille**				
St5	0.094 (0.015; 0.421)	0.071 (0.012; 0.329)	1.230 (0.839; 1.264)	1.324 (0.854; 1.685)
St2	0.231 (0.115; 0.410)	0.186 (0.100; 0.319])	1.011 (0.783; 1.157)	1.242 (0.898; 1.567)
**Elan**				
St3	0.160 (0.077; 0.303)	0.134 (0.070; 0.241])	1.034 (0.835; 1.169)	1.194 (0.912; 1.472)
St4	0.315 (0.183; 0.485)	0.267 (0.167; 0.399])	0.865 (0.659; 1.013)	1.180 (0.842; 1.498)

^a^ Estimation from the best model selected in capture-recapture analysis (Table S2). ^b^ Estimation obtained from Pradel formulation.

**Table 4:** Main patterns of PUUV microevolution, rodent population dynamics, rodent seroprevalence, and environment for each station.

	PUUV Diversity	Population Dynamics	Seroprevalence (SP)	Environment
**Elan Forest**				
**Station 3**	No genetic diversity over time	Less extinction over time, peaks some years, good survival	Lower SP than in Croix-Scaille forest with peak in 2003	A limited forest of broadleaves, surrounded by fields and roads
**Station 4**	No genetic diversity over time	Less extinction over time, peaks some years, good survival	Lower SP than at other stations	
**Croix-Scaille Forest**				
**Station 2**	Highest genetic diversity over time and at each time	Overall good survival and abundance similar to Elan forest but extinction some years	Very high SP every year bank voles are present	Oaks with conifer plots in a large forest massif with harsher weather conditions than in Elan
**Station 5**	Lower genetic diversity than station 2 and higher than Elan forest	Numerous extinctions, lowest survival, and low abundance	Highest SP every year bank voles are present	Coniferous plot in a large forest massif with harsher weather conditions than in Elan

## Data Availability

The data presented in this study are available within the article (included supplementary materials) and on request from the corresponding authors.

## Supplementary Materials

The following are available online at https://www.mdpi.com/article/10.3390/pathogens10091164/s1, Table S1: Dataset of 55 sequences with accession numbers, sampling year, station, and cluster, Table S2: Survival and seniority probabilities model selection.
