# Supplementary material for: Puumala Virus Variants Circulating in Forests of Ardennes, France: Ten Years of Genetic Evolution
Source: Pathogens. 2021 Sep 9;10(9):1164. doi: 10.3390/pathogens10091164 (PMC8472060; doi:10.3390/pathogens10091164)
Supplement: Supplementary file 1 [file pathogens-10-01164-s001.zip › Supplementary Materials/Supplementary -S1.pdf]

Table S1. Dataset of 55 sequences with accession numbers, sampling year, station and cluster

Isolate	GenBank accession number	Station	Sampling year	Cluster	Variant
11-06	MT003264	2	2000	B	8
72-18	MT003272	2	2003	B	10
64-7	MT003265	2	2003	B	8
101-150	MT003281	2	2005	B	12
102-48	MT003282	2	2005	B	11
95-118	MT003276	2	2005	B	11
95-134	MT003277	2	2005	B	11
155-34	MT003232	2	2008	B	7
155-14	MT003231	2	2008	C	13
155-43	MT003233	2	2008	C	13
155-44	MT003234	2	2008	C	13
159-11	MT003237	2	2008	C	13
159-32	MT003238	2	2008	C	13
159-45	MT003239	2	2008	C	13
159-52	MT003240	2	2008	C	13
167-4	MT003247	2	2008	C	13
167-6	MT003248	2	2008	C	13
175-1	MT003251	2	2009	C	13
175-5	MT003252	2	2009	C	13
155-42	MT003230	2	2008	D	2
155-47	MT003235	2	2008	D	2
159-9	MT003241	2	2008	D	2
163-4	MT003244	2	2008	D	4
167-2	MT003246	2	2008	D	2
179-14	MT003256	2	2009	D	9
179-18	MT003257	2	2009	D	2
179-1	MT003254	2	2009	D	3
179-25	MT003258	2	2009	D	2
179-28	MT003259	2	2009	D	2
179-4	MT003255	2	2009	D	3
69-2	MT003268	3	2003	A	1
69-40	MT003269	3	2003	A	1
73-28	MT003274	3	2003	A	1
73-4	MT003273	3	2003	A	1
103-47	MT003283	3	2005	A	1
96-53	MT003278	3	2005	A	1
144-16	MT003263	3	2007	A	1
180-78	MT003260	3	2009	A	1
66-29	MT003266	4	2003	A	1
66-70	MT003267	4	2003	A	1

70-80	MT003270	4	2003	A	1
78-39	MT003275	4	2003	A	1
100-55	MT003280	4	2005	A	1
104-5	MT003284	4	2005	A	1
71-64	MT003271	5	2003	B	6
98-17	MT003279	5	2005	B	8
162-25	MT003243	5	2008	B	5
166-1	MT003245	5	2008	B	5
170-3	MT003250	5	2008	B	5
178-2	MT003253	5	2009	B	5
189-3	MT003261	5	2009	B	5
189-4	MT003262	5	2009	B	5
158-3	MT003236	5	2008	D	2
162-13	MT003242	5	2008	D	2
170-1	MT003249	5	2008	D	2
