# Supplementary material for: Puumala Virus Variants Circulating in Forests of Ardennes, France: Ten Years of Genetic Evolution
Source: Pathogens. 2021 Sep 9;10(9):1164. doi: 10.3390/pathogens10091164 (PMC8472060; doi:10.3390/pathogens10091164)
Supplement: Supplementary file 1 [file pathogens-10-01164-s001.zip › Supplementary Materials/Supplementary -S2.pdf]

Table S2. Survival and seniority probabilities model selection. Columns represent Akaike Information Criterion with small sample correction (QAICc), QAICc differences (ΔQAICc), normalised QAICc weights (wi), and numbers of parameters (np). Terms (t), (.) and (station) denote parameterisations of ϕ, ϒ and p using models and represent respectively time variation, no time variation and variation with station, respectively. The selected models are highlighted in bold. Models with † and ‡ indicate null and general models respectively.

**Models**	**QAICc**	**ΔQAICc**	***wi***	**np**
**Survival rates**				
**ϕ (station) p (t)**	**395.10**	**0.00**	**0.70**	**13**
ϕ (.) p (t)	398.02	2.11	0.25	10
ϕ (station) p (station)	403.44	7.54	0.02	8
ϕ (station) p (.)	403.98	8.07	0.01	5
ϕ (t) p (.)	404.96	9.05	0.01	10
ϕ (.) p (.) †	405.08	9.17	0.01	2
ϕ (t) p (t)	406.15	10.24	0.00	17
ϕ (t) p (station)	407.83	11.92	0.00	13
ϕ (.) p (station)	408.11	12.20	0.00	5
ϕ (station*t) p (station)	420.64	24.73	0.00	37
ϕ (station*t) p (.)	422.12	26.22	0.00	35
ϕ (station) p (station*t)	426.12	30.22	0.00	40
ϕ (.) p (station*t)	426.55	30.64	0.00	37
ϕ (station*t) p (t)	427.16	31.25	0.00	42
ϕ (t) p (station*t)	436.36	40.45	0.00	44
ϕ (station*t) p (station*t) ‡	440.68	44.78	0.00	50
**Seniority probabilities**				
**ϒ (station) p (t)**	**379.25**	**0.00**	**0.81**	**13**
ϒ (.) p (t)	382.25	3.01	0.18	10
ϒ (t) p (.)	390.39	11.14	0.00	10
ϒ (t) p (t)	390.79	11.55	0.00	17
ϒ (t) p (station)	392.56	13.31	0.00	13
ϒ (station*t) p (t)	401.30	22.06	0.00	41
ϒ (station*t) p (.)	401.33	22.09	0.00	35
ϒ (station) p (station)	402.34	23.09	0.00	8
ϒ (station*t) p (station)	402.38	23.13	0.00	37
ϒ (station) p (.)	403.01	23.77	0.00	5
ϒ (.) p (.) †	405.20	25.95	0.00	2
ϒ (.) p (station)	407.46	28.21	0.00	5
ϒ (station) p (station*t)	408.77	29.53	0.00	40
ϒ (.) p (station*t)	410.45	31.20	0.00	37
ϒ (t) p (station*t)	415.92	36.68	0.00	44
ϒ (station*t) p (station*t) ‡	421.41	42.17	0.00	52
